# Subacute and Chronic Spinal Cord Injury: A Scoping Review of Epigenetics and Secondary Health Conditions

**DOI:** 10.1177/25168657231205679

**Published:** 2023-10-28

**Authors:** Letitia Y Graves, Kayla F Keane, Jacquelyn Y Taylor, Tzu-fang Wang, Leorey Saligan, Kath M Bogie

**Affiliations:** 1 School of Nursing, University of Texas Medical Branch, Galveston, TX, USA; 2 Louis Stokes Cleveland Veterans Affairs Medical Center, Cleveland, OH, USA; 3 National Institute of Nursing Research, National Institutes of Health, Bethesda, MD, USA; 4 Columbia School of Nursing and Center for Research on People of Color, New York, NY, USA; 5 National Cancer Institute, National Institutes of Health, Bethesda, MD, USA; 6 Case Western Reserve University, Cleveland, OH, USA

**Keywords:** Spinal cord injury, epigenetics, neuroepigenetics, scoping review

## Abstract

**Background:**

Epigenetics studies the impact of environmental and behavioral factors on stable phenotypic changes; however, the state of the science examining epigenomic mechanisms of regulation related to secondary health conditions (SHCs) and neuroepigenetics in chronic spinal cord injury (SCI) remain markedly underdeveloped.

**Objective:**

This scoping review seeks to understand the state of the science in epigenetics and secondary complications following SCI.

**Methods:**

A literature search was conducted, yielding 277 articles. The inclusion criteria were articles (1) investigating SCI and (2) examining epigenetic regulation as part of the study methodology. A total of 23 articles were selected for final inclusion.

**Results:**

Of the 23 articles 52% focused on histone modification, while 26% focused on DNA methylation. One study had a human sample, while the majority sampled rats and mice. Primarily, studies examined regeneration, with only one study looking at clinically relevant SHC, such as neuropathic pain.

**Discussion:**

The findings of this scoping review offer exciting insights into epigenetic and neuroepigenetic application in SCI research. Several key genes, proteins, and pathways emerged across studies, suggesting the critical role of epigenetic regulation in biological processes. This review reinforced the dearth of studies that leverage epigenetic methods to identify prognostic biomarkers in SHCs. Preclinical models of SCI were genotypically and phenotypically similar, which is not reflective of the heterogeneity found in the clinical population of persons with SCI. There is a need to develop better preclinical models and more studies that examine the role of genomics and epigenomics in understanding the diverse health outcomes associated with traumatic SCI.

**Clinical Relevance:** Identification of the mechanisms and relevant prognostic biomarkers that regulate the epigenome can support the development of targeted clinical decision making tools, and interventions. This article highlights the need to advance translational and precision applications in SCI research, bringing personalized care into the clinical environment.

## Introduction

Spinal cord injury (SCI) is a devastating condition that often yields permanent disability and challenges in medical care and symptom management. This once “ailment to not be treated” has over the years seen increased life expectancy resulting from advances in adaptive technology, pharmacologic intervention, and more robust understanding of SCI pathophysiology.^1^ The SCI population is highly heterogenous in both American Injury Scale (AIS) level and the extent of permanent neurologic deficit. No two patients will have an identical pathophysiologic progression or clinical course post injury.^2^ However, functional recovery continues to be hampered by a paucity of clinical treatment options. Regenerative approaches are ongoing with small progress; treatment strategies remain exploratory, and few have translated to patients (T2), practice(T3), or community (T4) on the translational research spectrum.^3^ Post-injury management of secondary health conditions (SHCs) and complications is the key to longevity with good health and acceptable quality of life.

There are 3 generally accepted phases of recovery following traumatic SCI: (1) acute (hours to days), (2) subacute (weeks to months), and (3) chronic (>1 year) states. However, it is also accepted that more than 6 months post-injury is also considered chronic and may be more commonly reflected in preclinical studies.^4^ The literature lacks consensus on how chronicity is defined likely due to the interindividual differences in functional recovery between human and preclinical pathophysiology. Thus, for the current review, we define chronicity as starting between 6 months to 1 year following injury.

It has been found that most of the research on SCI has focused on acute and chronic phases.^5^ This is likely to be due to the interest in time-dependent inflammatory and immune responses post trauma. There is also increasing recognition that the long-term impact of traumatic SCI may be characterized as a sub-clinical inflammatory disease. A review by Noller and colleagues^6^ discussed the contribution of persistent chronic inflammation in SCI resulting in common SHCs. Chronic inflammation is significant for individuals living with SCI because of the increased risk of cardiometabolic disorders that are the leading cause of morbidity and mortality in this population. Several researchers have shown SCI-specific inflammatory and structural markers found in the blood/cerebrospinal fluid (CSF) linked to injury and neurologic loss may be predictive of functional recovery in SCI.^7-10^

Epigenetics is broadly defined as processes that may regulate gene expression and do not involve changes to the underlying DNA sequence: a change in phenotype without a change in genotype.^11,12^ Disruption of gene expression patterns governed by hyper, or hypo methylation may result in autoimmune diseases, cancers, and various other illnesses, in contrast to genetic changes, which are difficult to reverse. However, environmental (social and structural) influences on DNA methylation may be reversed if reduced or eliminated (discrimination, stress, smoking, etc.), and pharmaceutical interventions have also been used to reverse effects.^13-16^ The field of epigenetic research seeks to discover how lived environment, social condition, psychosocial factors, and nutrition affect an individual’s expression of genetic information.^16^ The 3 central mechanisms regulating epigenetics are (a) alteration of histone modification, (b) DNA methylation (DNAm), and (c) RNA regulation.^17^

Neuroepigenetics is distinguished from classic epigenetics due to its specific application to neurons, which means modifications are not propagated to progeny cells as seen in non-neuronal cells.^18^ However, the central regulating mechanisms remain the same. As more is being understood about environmental influences on disease development, multimorbidity, and health outcomes, epigenetic methodology opens the door to leverage biological and clinical data to inform SHC phenotypes in persons with SCI. Hence, to better understand the evolving science of epigenetics and its application in SCI research, we conducted a scoping review.

Scoping reviews differ from systematic reviews in that they do not aim to produce a critically appraised and synthesized result/answer to a particular question, but rather focus on (1) identifying certain characteristics/concepts in papers or studies and (2) mapping, reporting, or discussing these characteristics/concepts.^19^ A scoping approach was chosen to guide this review to map and characterize studies investigating epigenetic regulation and their links to SHCs after acute and chronic SCI. We hypothesize that given the paucity of literature on this topic, the influence of epigenetics remains an understudied area for this population. The specific purpose of this scoping review was to examine the state of the science focusing on epigenetics that attempts to explain SHC and other conditions associated with chronic SCI.

## Methods

A librarian-assisted literature search was conducted across 4 databases (PubMed; Embase; Web of Science; Scopus) using the search terms “spinal cord injury,” “spinal cord injuries,” “epigenetics,” “epigenome,” “histone modification,” and “chromatin modification.” An Endnote library was shared with 277 articles from the years 2010 to 2020.

**Figure 1.**
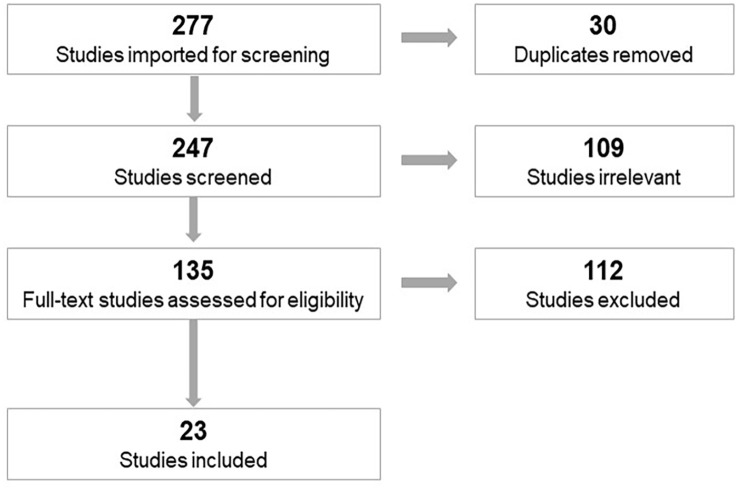
Prisma flow chart.

Covidence review software (v2472 44496257; Melbourne, Australia) was used to facilitate abstract and full text review. For inclusion, articles (1) investigated SCI and (2) examined a mechanism of epigenetic regulation as part of the study methodology. Articles were further excluded if they were reviews, commentaries, book chapters, or unavailable in English. Articles were uploaded to the Covidence software, and 30 duplicates were removed, resulting in 247 articles remaining for title and abstract review. Two reviewers completed the abstract review (author *LYG, T-FW*), and 135 studies were retained for full text review. Three reviewers completed the full text review and final article abstraction (author *LYG, T-FW, KFK*). One author served to resolve conflicts if they arose (author *LYG*). After the removal of articles that did not meet inclusion criteria, a total of 23 articles was selected for final inclusion (Figure 1).

The software did not offer Cohen’s Kappa; however, a pilot practice of 7 articles was done among full text reviewers to ensure that inter-rater reliability was consistent. No conflicts were found, and reviewer responses to screening tools were consistent across sections and articles.

## Results

Of the 23 articles included, 65% of studies examined mechanisms related to histone modification, with 26% of studies using DNA methylation (DNAm) and 9% using RNA regulation as epigenetic mechanisms. The studies occurred with mice (43%) and rats (44%) as the most commonly studied species. Only one human study was identified in this review. Most of the studies focused primarily on central nervous system (CNS) regeneration and plasticity, with only one article considered clinically relevant for SHC, such as neuropathic pain. We have divided and will discuss the included articles by epigenetic mechanism.

### DNA methylation (DNAm)

**Figure 2.**
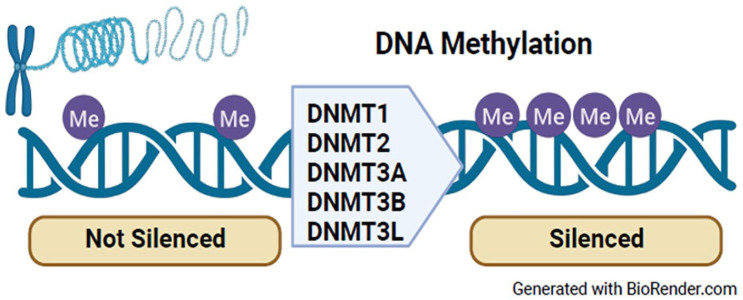
DNA methylation.

DNAm is the most well characterized and most common epigenetic mechanism studied, with a role in the regulation of transcription, chromatin structure, genomic imprinting, and chromosome stability.^20^ Methyltransferase enzymes (DNMT1, DNMT3A, and DNMT3B), are responsible for the transfer of the methyl group to DNA and are responsible for the mediation and maintenance of DNAm patterns (Figure 2).^20^ Of the 23 articles, only 6 studies focused on demonstrating the contribution of DNAm in the process of nerve regeneration and remodeling after SCI. Two of those articles looked at the role of folic acid/folate pathway as a key methyl donor in the CNS, for CNS regeneration and repair, and as a target to reduce neuropathic pain.^21,22^

In an earlier study by Iskandar et al^21^ an SCI regeneration model (dorsal column transection and conditioning sciatic nerve injury) was used to analyze changes in folate receptor (*Folr*) expression, global methylation, and methyltransferase levels in wild-type and *Folr* knock-out adult male Sprague-Dawley rats and male mice. They found that injury-induced augmentation of *Folr* receptor expression is important to the regeneration effects of folic acid on the spinal cord. Changes in the *Folr* expression were related to the suppression of donor methyl groups to enable synthesis of S-adenosylmethionine (*SAM*), the primary donor in neurons and all cells of the CNS.^21^ DNA methyltransferases (*Dnmts*) were directly involved in the process via the methylation of hemi-methylated sites by *Dnmt 1* and de novo methylation of previously unmethylated sites by *Dnmt3a* and *Dnmt3b*, resulting in detectable effects on suppressed *Dnmt3a* and *Dnmt3b* protein levels, returning to baseline following folic acid treatment. Global methylation decreased by 40% in the presence of injury (SCI) with the use of folic acid supplementation.

Miranpuri et al^22^ used a contusion SCI model in adult male Sprague-Dawley rates to analyze the effect of folic acid on matrix metalloproteinase 2 (*MMP2*) expression and neuropathic pain. *MMP2* is a protein coding gene found to be associated with diverse function, such as the induction of mitochondrial-nuclear stress signaling with activation of the pro-inflammatory *NF-κB, NFAT*, and *IRF* transcriptional pathways.^23^ They found that locomotor function improved among the folic-acid-treated group during mid and late phases of the injury, that is, comparable to subacute and chronic phases of SCI in humans, compared with the control (water) group. Moreover, those rats in the folic acid treatment group also demonstrated reduction in neuropathic pain compared to the control (water) group. In summary, folic acid and the folate pathway are mediated by epigenetic mechanisms. The use of folic acid supplementation appears to promote endogenous axonal regeneration and spinal cord healing.

Shi et al^24^ used Wistar rats to examine the role of abnormal DNAm in axonal regeneration and cell proliferation through histological observation, motor function assessment, and whole-genome bisulfate sequencing (WGBS) to detect gene methylation with Gene Ontology (GO) and KEGG pathway analyses. Histologically, they found several cavities, tissue necrosis, and glial scaring, with poor motor function in behavioral analysis. Bioinformatic analyses revealed 96 differentially methylated genes (DMG’s). Biological processes of the hypermethylated genes (50) were related to cell shape regulation and neurogenesis, while the biological processes enriched by the hypomethylated genes were related to brain development, protein phosphorylation, and ethanol response.

Another study by Shi et al^25^ reported results of a bioinformatic analysis of data to identify common hypermethylated and demethylated genes from DNAm sequencing of samples taken before and after sciatic nerve injury in rats.^25^ Although the focus of this paper examined common DMG’s among SCI and non-CNS injury (sciatic nerve injury), one area of interest is identifying protein-protein interaction networks specific to SCI that may have shared pathways significant to SCI-related SHC. The findings of this study indicated 12 DMGs that are involved in biological regulation processes, such as cellular signaling and molecular functions of protein binding. Of particular interest was the Hippo signaling pathway given its activation by many demethylated genes important to neuroregeneration.^25^

Sun et al,^26^ examined the perturbations of hydropymethylcytosine (5hmC), an oxidation product of DNAm, on gene expression related to cell death after SCI. In other neurological conditions such as TBI, stroke, and sporadic amyotrophic lateral sclerosis (ALS), 5hmC has been found to play an important role in ischemia and perfusion to the brain and spinal cord. A contusion SCI model was used in rats at the T9-T11, followed by quantitative real-time polymerase chain reaction analysis (qRT-PCR) and hydroxymethylated DNA immunoprecipitation (hMeDIP) assays. Results revealed that global change in DNA 5hmC levels significantly increased at 6, 12, and 24 hours after SCI. Additionally, gene expression changes were found to increase in the presence of ten-eleven translocation family (*Tet2*) enzyme and worsened SCI in the presence of *SC-1*, a *Tet2* expression suppressor, suggesting the importance of 5hmC in the expression of genes related to cell death and survival in SCI and other neurologic conditions.

Lastly, DNAm was used to examine the developing spinal cord and its response to environmental input during locomotor development related to hindlimb activity.^27^ Doherty et al^27^ focused on brain derived neurotrophic factor (*BDNF*), a known target of epigenetic modifications in the brain resulting from environmental influence. Male Sprague-Dawley pup rats underwent low-thoracic spinal cord transection and behavioral testing. This study was among the first to examine the epigenome of the developing spinal cord and the environmental influence on motor outcomes. The findings of this study support that SCI is linked to large increases in methylation throughout the spine.

### Histone modification

**Figure 3.**
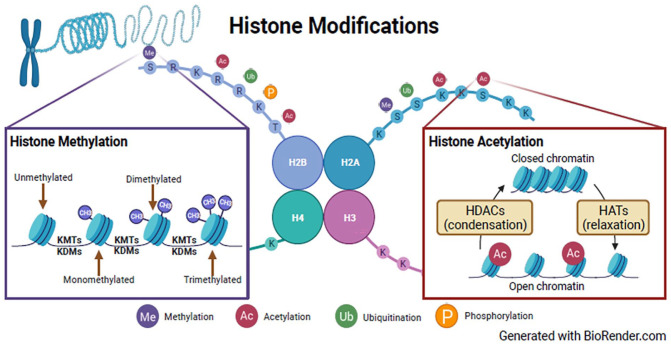
Histone modification.

The bulk of the studies examined histone modification, the covalent, post-translational modification of histones. This epigenetic regulatory process impacts gene expression by altering chromatin structure (phosphorylation, ubiquitination, acetylation, and methylation) or recruiting histone modifiers, such as histone acetyltransferase (HAT), histone methyl transferase (HMT), histone deacetylase (HDAC), histone demethylase (HDMT), kinases, and E3-ubiquitin (Figure 3).^28,29^ Six studies focused on histone acetylation, 6 examined histone deacetylation, and 3 examined histone demethylations. Histone acetylation is regulated by HAT and HDAC enzymes that add or remove acetyl groups to the N-terminal histone tails and together maintain acetylation homeostasis and play a role in plasticity transcription and regeneration-associated genes.^30^ Evidence demonstrates that downregulation of HDACs has positive effects on learning, memory, and synaptic plasticity. Seira and colleagues^31^ assessed the potential effect of trichostatin A (*TSA*), a known promotor of synaptic plasticity, neurogenesis, neuroprotection, and neurite branching in *Pten* knockout mice. They found that *TSA* had inhibitory effectors on HDAC, allowing for more acetylation and supporting axonal regeneration in young mice with *Pten* deletion.

De Menezes et al^32^ measured glial fibrillary acidic protein (*GFAP*), S100 calcium-binding protein B (*S100B*), and global histone h4 levels in Wistar rats. They found that global histone H4 acetylation levels in the perilesional tissue are time-related after SCI, with the 72-hour (acute) timepoint revealing an increase in global histone H4 acetylation levels.

Hutson et al^33^ examined the influence of environmental enrichment versus standard housing (control) on Cbp-dependent histone acetylation-mediated axon regeneration. Enriched environment (EE) offers opportunities for increased motor, social, and sensory activity and has been found to enhance neural connections and improve functional recovery on behavioral testing.^34,35^ They found that by exposing mice to EE, cAMP-response element binding protein had a mediation effect on histone acetylation; thus, changes in chromatin that increase regenerative capacity of neurons result in increased sensory and motor functions.^33^

The only human study in this scoping review was conducted by Goldhardt et al^36^ They examined the impact of a single body weight support gait training session on oxidative stress parameters, *BDNF* modulation, and global histone H3 and H4 acetylation levels in SCI participants. No changes were observed in BDNF levels, nor histone acetylation levels; however, there was an observed increase in levels of oxidative stress markers (*AOPPs*, nitrite, and *TBARSs*) in both treadmill and walker groups.

Rudman et al^37^ examined the role of bromodomain and extraterminal (*BET*) domain-containing proteins (epigenetic readers that bind acetylated lysines) on neuroinflammation after contusive SCI. The first finding was that BETs were expressed in all major CNS cell populations and their mRNA levels were not modulated in response to SCI. They also found that *BET* inhibition (via treatment with *BET* inhibitor JQ1) attenuated pro-inflammatory cytokine expression in activated primary CNS cells, astrocytes, oligodendrocytes, and microglia. Sánchez-Ventura et al^38^ also found that BET inhibition was effective in reducing pro-inflammatory cytokine production, promoting neuroprotection, and reducing acute neuropathic pain in a mouse contusion model.

Abdanipour et al^39^ tested valproic acid (VPA), a known HDAC inhibitor, in rats after SCI. HDAC inhibition results in transcriptional reactivation by inducing histone acetylation through HDAC enzyme suppression. Their findings support earlier studies which also report neuroprotective effects of VPA and increased *BDNF* and *GDNF* mRNA levels in neurodegenerative conditions.^39^ Kim et al^40^ investigated changes in the expression of the main regulators of neuronal survival and death. Following contusion injury to young male mice, they found the upregulation of *BDNF*, *GDNF*, and *HDAC1* expressions, with *HDAC1* being an important regulator of neuron apoptosis. Another study of changes in the main regulators of neuronal development, survival, and death in a contusion SCI rat found similar results.^41^ Neurotrophic factors *BDNF, NGG, GDNF*, and *HDAC1* were examined, and *BDNF* was found to be significantly elevated in the SCI group compared to the control, while the other factors were not found highly expressed. Although increased levels of HDACs were found to be expressed in the brain, their role in brain reorganization after injury remains poorly understood.

Qi and Wang^42^ examined class IIa HDACs, which differ from class I and class IIb HDACs by their sequence and structural organization, in that they rarely interact with histone tails or the removal of acetylated lysine groups. To better understand the epigenetic mechanism of macrophages (used to transition from phenotype M1 associated with pro-inflammation to phenotype M2 found to suppress excess inflammation, prevent axonal damage, and improve locomotor function), a male mice contusion model was tested with *TMP269*, a highly selective class IIa HDAC inhibitor. They found that the *TMP269*-treated group had higher levels of pro-inflammatory cytokines (IL-6 and TNF alpha), suggesting the critical role of class IIa HDACs as powerful inhibitors of pro-inflammatory cytokines in macrophages. Sanchez et al specifically examined HDAC3. They hypothesized that HDAC3 inhibition suppresses the phenotype M1 (pro-inflammatory) and promotes the anti-inflammatory phenotype M2 to improve functional SCI recovery. By introducing scriptaid and *RGFP966*, known HDAC3 inhibitors to female mice with T-cut spinal cord hemisection injury, they found that histone 3 and 4 acetylation were increased, and macrophage polarization was skewed toward M2 phenotype (anti-inflammatory).^43^ Scriptaid treatment also decreased M1 macrophage foaminess, while *RGFP966* significantly reduced the formation of foamy macrophages in both. They also found that foamy macrophages became proinflammatory and hindered regeneration. Taken together, HDAC inhibitors are strong targets for macrophage polarization and creating an anti-inflammatory environment for SCI recovery.

Hervera et al^44^ also studied HDAC3 to determine its role as a novel mechanism to discriminate between axonal regeneration and regenerative failure in dorsal root ganglia (DRG) sensory neurons. Conducting both in vivo and bioinformatic analysis, they found that reduced HDAC3 activity allowed for increased histone acetylation and regenerative gene expression in DRG. However, HDAC3 phosphorylation is calcium dependent and spinal injury does not induce the calcium-dependent protein phosphate 4 (*PP4)* activity needed for this process. The finding of both analyses supports the role of HDAC3 in restricting the regeneration program needed to enhance DRG neurite growth and expression of regeneration-associated genes including *JUN, MYC, STAT3, ATF3, and FOS* with key signaling pathways of MAPK, insulin, and JAK-STAT.^44^

Lee et al^45,46^ and Ni et al^47^ investigated the epigenetic regulation of angiogenesis and vascular regeneration in SCI. The study of post-SCI vascular regeneration by Ni et al ^47^ examined mRNA levels of *UTX, EZH2, Jmjd3, CBX8, CBX4, CBX6, and CBX7* at the site of the spinal lesion post-SCI contusion in C57BL/6J wild-type and *UTX* knockout male mice. They found that *UTX* was elevated at the day 3 timepoint and remained elevated at days 7 and 14 and was found to occur in CD31+ endothelial cells, implicating the potential role of *UTX* in vascular regeneration post SCI. They went on to demonstrate that *UTX* regulated expression of miR-24 (increased expression during myogenesis) by binding to the promoter and directly decreasing the level of methylation in several CpG positions in the miR-24 promoter, thereby promoting the expression of miR-24. In summary, *UTX* deletion can epigenetically promote vascular regeneration and functional recovery post SCI via a regulatory network with miR-24.

In both Lee et al studies,^45,46^ histone H3K27 demethylation *Jmjd3* was characterized for its role in acute inflammatory response regulation. Lee et al^45^ used adult Sprague Dawley male rats subjected to T10 contusion. They found that *Jmjd3* gene expression was increased within the first 8 hours after SCI and was more highly expressed in blood vessels at or near the injured spinal cord lesion site. This suggested that it is acutely upregulated following injury with specific functions in acute immune response and/or the blood-spinal cord barrier (BSCB). Moreover, given its role as a transcription activator through demethylation of trimethylated histone H3K27, it is also associated with inflammation-specific markers. The Lee et al^45^ study found that IL-6 showed a similar upregulation pattern as Jmjd3, which indicated that in the absence of Jmjd3, the upregulation of *IL-6* is inhibited. Lastly, transcription activators known to bind to *IL-6* gene promoters, such as *NF-kB*, were examined for interaction with *Jmjd3* using both Western blot and chromatin immunoprecipitation (ChIP) assays. They found that *Jmjd3* was directly involved in IL-6 gene activation by demethylation of H3K27me3 at the promoter in cooperation with *NF-kB* and *C/EBP*.^45^

Lee et al^46^ contribute findings to the critical role of histone H3K27 demethylase *Jmjd3* in the regulation of matrix metalloprotease (*MMP*) gene expression and BSCB integrity. *MMP* activation is known to contribute to permanent neurological disability after SCI because of blood cell infiltration, inflammation, and apoptosis.^45^ *MMP-2, MMP-3*, and *MMP-9 are* specifically known to be involved in SCI-induced BSCB disruption, but the mechanisms following SCI are not well understood.^45^ Again, using adult Sprague-Dawley male rats, SCI was induced using a contusion model at T9-T10. They found that *Jmjd3* activated *MMP-2, MMP-3*, and *MMP-9* expression in brain endothelial cells upon OGD/reperfusion injury. Using *Jmjd3* siRNA to deplete the *Jmjd3* transcript, they determined that depletion of *Jmjd3* expression inhibited the upregulation of *MMP-2, MMP-3*, and *MMP-9* expression and activities. Like their previous work that found *NF-kB* to be associated with Jmjd3 activation of *IL-6* gene expression, they found in this study that *NF-kB* was also required for *Jmjd3*-mediated *MMP-2, MMP-3*, and *MMP-9* gene activations. These 2 studies reinforce the significance of *NF-kB* involvement as a mediator of gene expression both in DNA methylation and histone demethylation. The combined results of all 3 studies provide potential therapeutic clues for targeting vascular regeneration in the treatment of SCI.

### RNA regulation

**Figure 4.**
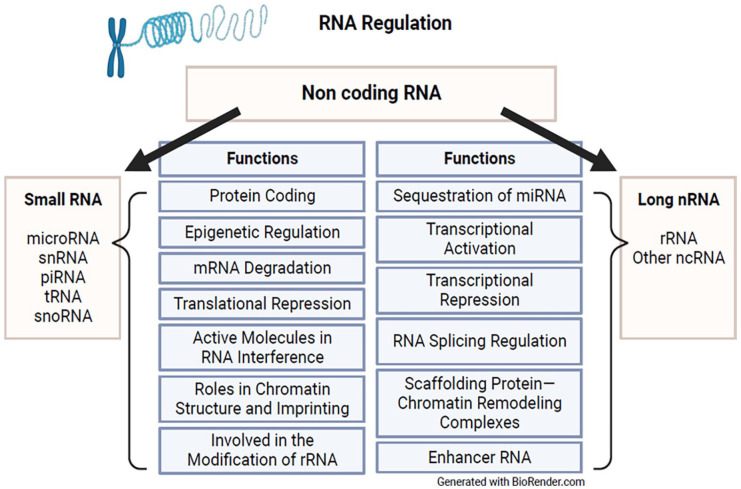
RNA regulation.

miRNA is a class of non-coding small RNA that functions to downregulate gene expression through mRNA cleavage, translational inactivation, and deadenylation (Figure 4).^16^ Using small interfering RNAs (siRNAs) to inhibit the interphase phase of the cell cycle, Wang et al^48^ studied epigenetic silencing of cyclinD1 in bone-marrow-derived mesenchymal stem cells (BMSCs) for protective effects in rats after SCI. Cyclins are proteins that function to progress the cell cycle by binding a group of enzymes called cyclin-dependent kinases (CDKs) which phosphorylate target proteins.^49^ CyclinD1 specifically is responsible for progressing the cell cycle from G1 to S phase.^50^ Inhibition of CyclinD1 expression is associated with downregulation of pro-inflammatory cytokines,^50^ which may be a potential therapeutic target for pro-inflammatory conditions such as SCI.

In female Sprague Dawley rats, cyclinD1 was transplanted with BMSCs into the lesion area of the SCI using siRNA plasmids. CyclinD1 was found nominally expressed by Western blotting and qRT-PCR, confirming that siRNA transfection was successful. Observations of histological changes confirmed decreased tissue edema, inflammatory cell infiltration at the site of the SCI lesion, and smaller syrinx in groups with si-cyclinD1 + BMSCs and BMSC groups compared with the control and sham groups. Additionally, *GFAP*-positive and *NGF*-positive cells were significantly increased in the si-cyclinD1 + BMSCs and BMSC groups compared with the control group. *GFAP* and *NGF* regulation were associated with enhanced astrocyte proliferation. These findings, along with higher BBB scores in groups with si-CyclinD1-engineered BMSCs, support that by silencing CyclinD1, BMSC may promote functional recovery.

In another study investigating the subacute phase of SCI, Wang et al ^48^ sought to use bioinformatic analysis to construct and integrate ceRNA network of critical genes, miRNAs, long non-coding RNAs (lncRNA), transcription factors, and signaling pathways. Gene expression data sets were used from the gene expression omnibus (GEO) database to define the “SCI group” as mice with SCI acquired by contusion. DEG analysis was performed using GO and KEGG enrichment. They found 4 genes (*Flna, Id3, Hk2, and Ywhae*), 4 transcription factors (Sp1, Trp53, Jun, and RelA), 2 miRNAs (miR-16-5p and miR-325-3p), and 3 lncRNAs (Neat1, Xist, Malat1) that are considered significant contributors to the pathogenesis of subacute SCI.^48^

## Discussion

The results of this scoping review suggest that there is an increasing enthusiasm for epigenetics and neuroepigenetics in SCI research, especially given the increased complexity when considering the different cell types that exist in the brain and spine. Further, the findings from this review offer exciting insights into neural circuits and synaptic plasticity involved in learning new information. The emerging sub-discipline of neuroepigenetics describes the “discoveries of a wide variety of roles for epigenetic molecular mechanisms in the central nervous system (CNS) regarding acquired behaviors, CNS disorders, neural plasticity, neurotoxicity, and drug addiction.”^51^ Current evidence suggests that in the presence of lost neuronal acetylation homeostasis, short- and long-term chromatin architecture changes, resulting in the development of neurodegenerative disorders, such as Parkinson’s disease (PD), multiple sclerosis (MS), stroke (CVA), and Alzheimer’s disease (AD), which has significance post-SCI neurodegenerative changes.^52^ Notably, the histone acetylation-deacetylation balance modulates different cell types, such as inflammatory and immunological cells, which influence the severity of blood-brain barrier dysfunction, axonal demyelination, oxidative stress, and injury impact.^53,54^ Applying neuroepigenetics to SCI research could lead to new approaches in rehabilitation where re-learning motor activities could improve functional outcomes.

This scoping review also reinforces the dearth of studies that examine the potential of clinically applied precision health within the rehabilitation setting, particularly for individuals living with SCI and managing SCI-associated SHCs. Many of the studies included in this review examined changes in the acute or subacute post-SCI phase. None of the studies looked at these gene expression changes in chronic SCI, thus leaving a larger gap in the significance of chronicity on these mechanisms. Understanding gene expression and its regulation translates to more accurate diagnosis and treatment for clinically significant changes in phenotype. Wagner^55^ presented the Rehabilomics research framework, offering a translational path for rehabilitation research that combines omics technology with personal biology, physiological responses to environmental exposures, and biopsychosocial outcomes based on the World Health Organization International Classification of Functioning, Disability, and Health domains. However, this adapted model primarily focuses its application on traumatic brain injury (TBI) research. While TBI and SCI share similarities in primary and secondary injury cascades following mechanical injury, the study of SCI and the chronic SHCs that follow remain understudied. Individuals living with SCI are living longer and have increased and/or unpredictable levels of risk for developing a wide range of SCI-driven SHCs. The identification and use of prognostic biomarkers are major steps in developing decision trees, predictive tools, personalized interventions, and treatments.

### Key genes, proteins, pathways

The subacute phase after SCI is categorized by neuronal apoptosis, axonal demyelination, Wallerian degeneration, axonal remodeling, and glial scar formation, while the chronic phase is characterized by cystic cavity, axonal dieback, and maturation of glial scar.^3^ These clinical features are reinforced by the findings of studies included in this scoping review, particularly as key genes, proteins, and pathways that were present in multiple studies, including brain derived neurotrophic factor (*BDNF*), glial cell derived neurotrophic factor (*GDNF*), neuronal growth factor (*NGF*), histone deacetylase 1 (*HDAC1*), NF-kappaB signaling pathway, and the Janus kinase-signal transducer and activator of transcription (JAK-STAT) pathways. These key genes and pathways offer an opportunity to develop our understanding of mechanisms underlying SCI-triggered SHC that result from gene expression activation and/or silencing.

*BDNF* is well known for its glucose and energy metabolism regulation, as well as neuroplasticity and synaptogenesis. The JAK-STAT pathway is an essential signaling pathway for several cellular response functions including inflammatory responses, hypoxia/reperfusion, cellular stress, and more.^56^ Saligan^57^ found that the *BDNF Val66MET* single nucleotide polymorphism (SNP) genotype was protective against cancer-related fatigue. This could be a significant target in the fatigue experienced by individuals living with SCI and chronic multimorbidity. *BDNF* is also implicated in stress-related phenotypes, which given its role in brain-based gene regulation, has implications for shared mechanisms of other stress-related conditions, such as depression and anxiety.^58^ *GDNF* is a protein coding gene specifically associated with SCI and is also involved in the RAF/MAP kinase cascade super pathway that, like JAK-STAT, regulates cellular processes related to differentiation, survival, senescence, and cell movement.

Preliminary work by Bogie and colleagues,^59,60^ has shown the genetic influence of candidate DNA variants and single nucleotide polymorphism (SNPs) on intramuscular adipose tissue (IMAT) accumulation and recurrent pressure injury (PrI) risk. They have previously shown that varying levels of circulatory biomarkers may be indicative of recurrent PrI risk.^59,60^ Furthermore, genetic biomarkers, specifically those related to fatty metabolism, are indicative of persons with SCI at highest risk for recurrent PrI.^61^ Histone H3K27me3 demethylase Jmjd3 has been found to be involved in wound repair and the inflammation process,^62,63^ highlighting the value of incorporating both genetic and epigenetic regulation of gene expression, as it may hold significance for the study of pressure injury and other SHCs after SCI. Thus, defining the epigenetic mechanisms linked to remodeling neural tissue would help researchers design therapeutic strategies to promote wound healing and tissue functionality after SCI among other potential targets.^64,65^

### Chronic multimorbidity in SCI research

There is a key discussion absent in SCI research around epigenetics, chronic multimorbidity, and SHCs. Symptom science is “focused on quantifying subjective symptom experiences and measuring the biologic, physiologic, and omics underpinnings of symptoms and sequela common to health conditions and their treatments”^66^ and recognizes the “complex relationships within and between symptoms both in a single chronic condition and among chronic conditions (ie, shared symptom cluster).”^67^ Symptoms serve as critical clues to changes in health status (physical and biopsychological) and are the primary reason urgent or emergent care is sought, which may (or may not) lead to hospitalization and/or diagnosis of a newly developed SHC. This is of particular importance for SCI research, where individuals are likely to suffer higher rates of multimorbid SHCs and the management of accompanying symptoms.

Diseases can often be due to multiple factors including routine environmental exposures.^68^ While we present the effects of epigenetics leading to the development of multimorbid SHC, that is, the gene-environment, it is very probable that multimorbid SHC directly lead to remodeling in the epigenome. Studies looking at the influence of changes due SHC on the epigenome need to address the dynamic variability inherent in epigenetic studies. Prospective studies to measure and compare pre-multimorbidity states are also challenging. Multimorbidity as previously described carries a significant burden for individuals living with SCI and is influenced by age and injury characteristics (ie, paraplegia; tetraplegia; etc.). These influences also carry significant complexity in the presence of the injury. Although the relationship between multimorbid SHC and epigenetic change is even less understood given the dearth of research in this field, we wanted to acknowledge it as an important consideration as more work is being done to develop this field.

Theoretical models have been developed to improve our understanding of the mechanisms that contribute to symptom behavior (ie, initiation, severity, temporality). However, symptom behavior and associated mechanisms has primarily been evaluated in other conditions, such as cancer and cardiovascular disease; only one study addresses symptom behavior in SCI research.^69-72^ Development of a Rehabilomics theoretical model specific to SCI symptomology would provide guidance that would support the translation of bench findings to clinical management of chronic multimorbidity resulting from SCI.

## Conclusion/Implications for Research and Practice

This scoping review has revealed some key epigenetic regulators of gene expression and adjacent pathways that appear to overlap many critical biological, molecular, and cellular processes following SCI. However, it also elucidates the need for more translational/applied clinical research as a next step from the bench to begin impacting the patient. Given the paucity of research that studies the concepts of epigenetics, SHCs, and/or their associated symptoms in SCI research (individual or integrated), more work in this area is critical to continue advancing our understanding of SCI-specific biobehavioral outcomes.

## References

[1] Donovan WH. Donald Munro Lecture. Spinal cord injury–past, present, and future. J Spinal Cord Med. 2007;30:85-100. 17591221 10.1080/10790268.2007.11753918 PMC2031949

[2] Albayar AA Roche A Swiatkowski P , et al. Biomarkers in spinal cord injury: prognostic insights and future potentials. Front Neurol. 2019;10:27. 30761068 10.3389/fneur.2019.00027 PMC6361789

[3] Anjum A Yazid MD Fauzi Daud M , et al. Spinal cord injury: pathophysiology, multimolecular interactions, and underlying recovery mechanisms. Int J Mol Sci. 2020;21:7533. 33066029 10.3390/ijms21207533 PMC7589539

[4] Tashiro S Nakamura M Okano H. Regenerative rehabilitation and stem cell therapy targeting chronic spinal cord injury: a review of preclinical studies. Cells. 2022;11:685. 35203335 10.3390/cells11040685 PMC8870591

[5] Ahuja CS Wilson JR Nori S , et al. Traumatic spinal cord injury. Nat Rev Dis Primers. 2017;3:17018. 28447605 10.1038/nrdp.2017.18

[6] Noller CM Groah SL Nash MS. Inflammatory stress effects on health and function after spinal cord injury. Top Spinal Cord Inj Rehabil Summer. 2017;23:207-217. 10.1310/sci2303-207 PMC5562028 29339896

[7] Kwon BK Streijger F Fallah N , et al. Cerebrospinal fluid biomarkers to stratify injury severity and predict outcome in human traumatic spinal cord injury. J Neurotrauma. 2017;34:567-580. 27349274 10.1089/neu.2016.4435

[bibr8-25168657231205679] Moghieb A Bramlett HM Das JH , et al. Differential neuroproteomic and systems biology analysis of spinal cord Injury. Mol Cell Proteomics. 2016;15:2379-2395. 27150525 10.1074/mcp.M116.058115 PMC4937511

[bibr9-25168657231205679] Sengupta MB Basu M Iswarari S , et al. CSF proteomics of secondary phase spinal cord injury in human subjects: perturbed molecular pathways post injury. PLoS One. 2014;9:e110885. 10.1371/journal.pone.0110885 PMC4211693 25350754

[10] Sharif S Jazaib Ali MY. Outcome Prediction in spinal cord injury: myth or reality. World Neurosurg. 2020;140:574-590. 32437998 10.1016/j.wneu.2020.05.043

[11] Campbell RR Wood MA. How the epigenome integrates information and reshapes the synapse. Nat Rev Neurosci. 2019;20:133-147. 30696992 10.1038/s41583-019-0121-9 PMC7032043

[12] Wang KC Chang HY. Epigenomics: technologies and applications. Circ Res. 2018;122:1191-1199. 29700067 10.1161/CIRCRESAHA.118.310998 PMC5929475

[13] Barcelona de Mendoza V Huang Y Crusto CA Sun YV Taylor JY. Perceived racial discrimination and DNA methylation among African American women in the InterGEN Study. Biol Res Nurs. 2018;20(2):145-152. 29258399 10.1177/1099800417748759 PMC5741522

[bibr14-25168657231205679] Klebaner D Huang Y Hui Q , et al. X chromosome-wide analysis identifies DNA methylation sites influenced by cigarette smoking. Clin Epigenetics. 2016;8:20. 26913089 10.1186/s13148-016-0189-2 PMC4765206

[bibr15-25168657231205679] Wright ML Huang Y Hui Q , et al. Parenting stress and DNA methylation among African Americans in the InterGEN Study. J Clin Transl Sci. 2017;1:328-333. 29707254 10.1017/cts.2018.3 PMC5915805

[16] Zhang L Lu Q Chang C. Epigenetics in health and disease. Adv Exp Med Biol. 2020;1253:3-55. 32445090 10.1007/978-981-15-3449-2_1

[17] Al Aboud NM Tupper C Jialal I. Genetics, Epigenetic Mechanism. [Updated 2023 August 14]. In: StatPearls [Internet]. StatPearls Publishing; 2023 January-. https://www.ncbi.nlm.nih.gov/books/NBK532999/30422591

[18] Hwang JY Aromolaran KA Zukin RS. The emerging field of epigenetics in neurodegeneration and neuroprotection. Nat Rev Neurosci. 2017;18:347-361. 28515491 10.1038/nrn.2017.46 PMC6380351

[19] Munn Z Peters MDJ Stern C Tufanaru C McArthur A Aromataris E. Systematic review or scoping review? Guidance for authors when choosing between a systematic or scoping review approach. BMC Med Res Methodol. 2018;18:143. 30453902 10.1186/s12874-018-0611-x PMC6245623

[20] Meng H Cao Y Qin J , et al. DNA methylation, its mediators and genome integrity. Int J Biol Sci. 2015;11:604-617. 25892967 10.7150/ijbs.11218 PMC4400391

[21] Iskandar BJ Rizk E Meier B , et al. Folate regulation of axonal regeneration in the rodent central nervous system through DNA methylation. J Clin Investig. 2010;120:1603-1616. 20424322 10.1172/JCI40000 PMC2860927

[22] Miranpuri GS Meethal SV Sampene E , et al. Folic acid modulates matrix metalloproteinase-2 expression, alleviates neuropathic pain, and improves functional recovery in spinal cord-Injured Rats. Ann Neurosci. 2017;24:74-81. 28588362 10.1159/000475896 PMC5448437

[23] Genecards: The Human Gene Database. MMP2 Gene—Matrix Metallopeptidase 2. Accessed February 22, 2023, https://www.genecards.org/cgi-bin/carddisp.pl?gene=MMP2&keywords=MMP2

[24] Shi GD Zhang XL Cheng X , et al. Abnormal DNA methylation in thoracic spinal cord tissue following transection injury. Med Sci Monit. 2018;24:8878-8890. 30531681 10.12659/MSM.913141 PMC6295140

[25] Shi G Zhou X Wang X Zhang X Zhang P Feng S. Signatures of altered DNA methylation gene expression after central and peripheral nerve injury. J Cell Physiol. 2020;235:5171-5181. 31691285 10.1002/jcp.29393

[26] Sun H Miao Z Wang H , et al. DNA hydroxymethylation mediated traumatic spinal injury by influencing cell death-related gene expression. J Cell Biochem. 2018;119:9295-9302. 30074258 10.1002/jcb.27200

[27] Doherty TS Bozeman AL Roth TL Brumley MR. DNA methylation and behavioral changes induced by neonatal spinal transection. Infant Behav Dev. 2019;57:101381. 31557646 10.1016/j.infbeh.2019.101381 PMC6878986

[28] Handy DE Castro R Loscalzo J. Epigenetic modifications: basic mechanisms and role in cardiovascular disease. Circulation. 2011;123:2145-2156. 21576679 10.1161/CIRCULATIONAHA.110.956839 PMC3107542

[29] Neganova ME Klochkov SG Aleksandrova YR Aliev G. Histone modifications in epigenetic regulation of cancer: perspectives and achieved progress. Semin Cancer Biol. 2022;83:452-471. 32814115 10.1016/j.semcancer.2020.07.015

[30] Saha RN Pahan K. HATs and HDACs in neurodegeneration: a tale of disconcerted acetylation homeostasis. Cell Death Differ. 2006;13:539-550. 16167067 10.1038/sj.cdd.4401769 PMC1963416

[31] Seira O Wang W Lee S Roskams J Tetzlaff W. HDAC inhibition leads to age-dependent opposite regenerative effect upon PTEN deletion in rubrospinal axons after SCI. Neurobiol Aging. 2020;90:99-109. 32171589 10.1016/j.neurobiolaging.2020.02.006

[32] de Menezes MF Nicola F Vital da Silva IR , et al. Glial fibrillary acidic protein levels are associated with global histone H4 acetylation after spinal cord injury in rats. Neural Regen Res. 2018;13:1945-1952. 30233068 10.4103/1673-5374.239443 PMC6183034

[33] Hutson TH Kathe C Palmisano I , et al. Cbp-dependent histone acetylation mediates axon regeneration induced by environmental enrichment in rodent spinal cord injury models. Sci Transl Med. 2019;11: eaaw2064. 10.1126/scitranslmed.aaw2064 PMC7355732 30971452

[34] Alwis DS Rajan R. Environmental enrichment and the sensory brain: the role of enrichment in remediating brain injury. Front Syst Neurosci. 2014;8:156. 25228861 10.3389/fnsys.2014.00156 PMC4151031

[35] Nithianantharajah J Hannan AJ. Enriched environments, experience-dependent plasticity and disorders of the nervous system. Nat Rev Neurosci. 2006;7:697-709. 16924259 10.1038/nrn1970

[36] Goldhardt MG Andreia A Dorneles GP , et al. Does a single bout of exercise impacts BDNF, oxidative stress and epigenetic markers in spinal cord injury patients? Funct Neurol. 2019;34:158-166. 32453997

[37] Rudman MD Choi JS Lee HE Tan SK Ayad NG Lee JK. Bromodomain and extraterminal domain-containing protein inhibition attenuates acute inflammation after spinal cord injury. Exp Neurol. 2018;309:181-192. 30134146 10.1016/j.expneurol.2018.08.005

[38] Sánchez-Ventura J Amo-Aparicio J Navarro X Penas C. BET protein inhibition regulates cytokine production and promotes neuroprotection after spinal cord injury. J Neuroinflammation. 2019;16:124. 31186006 10.1186/s12974-019-1511-7 PMC6560758

[39] Abdanipour A Schluesener HJ Tiraihi T. Effects of valproic acid, a histone deacetylase inhibitor, on improvement of locomotor function in rat spinal cord injury based on epigenetic science. Iran Biomed J. 2012;16:90-100. 22801282 10.6091/ibj.1060.2012 PMC3600951

[40] Kim JH Kim SH Cho SR , et al. The modulation of Neurotrophin and epigenetic regulators: implication for astrocyte proliferation and neuronal cell apoptosis after spinal cord injury. Ann Rehabil Med. 2016;40:559-567. 27606261 10.5535/arm.2016.40.4.559 PMC5012966

[41] Park BN Kim SW Cho SR Lee JY Lee YH Kim SH. Epigenetic regulation in the brain after spinal cord injury: a comparative study. J Korean Neurosurg Soc. 2013;53:337-341. 24003367 10.3340/jkns.2013.53.6.337 PMC3756125

[42] Qi X Wang P. Class IIa HDACs inhibitor TMP269 promotes M1 polarization of macrophages after spinal cord injury. J Cell Biochem. 2018;119:3081-3090. 29077222 10.1002/jcb.26446

[43] Sanchez S Lemmens S Baeten P , et al. HDAC3 inhibition promotes alternative activation of macrophages but does not affect functional recovery after spinal cord injury. Exp Neurobiol. 2018;27:437-452. 30429652 10.5607/en.2018.27.5.437 PMC6221838

[44] Hervera A Zhou L Palmisano I , et al. PP4-dependent HDAC3 dephosphorylation discriminates between axonal regeneration and regenerative failure. EMBO J. 2019;38:e101032. 10.15252/embj.2018101032 PMC6600644 31268609

[45] Lee JY Na WH Choi HY Lee KH Ju BG Yune TY. Jmjd3 mediates blood-spinal cord barrier disruption after spinal cord injury by regulating MMP-3 and MMP-9 expressions. Neurobiol Dis. 2016;95:66-81. 27425890 10.1016/j.nbd.2016.07.015

[46] Lee K Na W Lee JY , et al. Molecular mechanism of jmjd3-mediated interleukin-6 gene regulation in endothelial cells underlying spinal cord injury. J Neurochem. 2012;122:272-282. 22578249 10.1111/j.1471-4159.2012.07786.x

[47] Ni S Luo Z Jiang L , et al. UTX/KDM6A deletion promotes recovery of spinal cord injury by epigenetically regulating vascular regeneration. Mol Ther. 2019;27:2134-2146. 31495776 10.1016/j.ymthe.2019.08.009 PMC6904668

[48] Wang N He L Yang Y , et al. Integrated analysis of competing endogenous RNA (ceRNA) networks in subacute stage of spinal cord injury. Gene. 2020;726:144171. 31669638 10.1016/j.gene.2019.144171

[49] Khan Academy. Cell Cycle Regulators. Khan Academy. Accessed August 8, 2023. https://www.khanacademy.org/science/ap-biology/cell-communication-and-cell-cycle/regulation-of-cell-cycle/a/cell-cycle-regulators

[50] Wang Y Kong QJ Sun JC , et al. Protective effect of epigenetic silencing of CyclinD1 against spinal cord injury using bone marrow-derived mesenchymal stem cells in rats. J Cell Physiol. 2018;233:5361-5369. 29215736 10.1002/jcp.26354

[51] Sweatt JD. The emerging field of neuroepigenetics. Neuron. 2013;80:624-632. 24183015 10.1016/j.neuron.2013.10.023 PMC3878295

[52] Shukla S Tekwani BL. Histone deacetylases inhibitors in neurodegenerative diseases, neuroprotection and neuronal differentiation. Front Pharmacol. 2020;11:537. 32390854 10.3389/fphar.2020.00537 PMC7194116

[53] Faraco G Pancani T Formentini L , et al. Pharmacological inhibition of histone deacetylases by suberoylanilide hydroxamic acid specifically alters gene expression and reduces ischemic injury in the mouse brain. Mol Pharmacol. 2006;70:1876-1884. 16946032 10.1124/mol.106.027912

[54] Chuang DM Leng Y Marinova Z Kim HJ Chiu CT. Multiple roles of HDAC inhibition in neurodegenerative conditions. Trends Neurosci. 2009;32:591-601. 19775759 10.1016/j.tins.2009.06.002 PMC2771446

[55] Wagner AK. TBI rehabilomics research: an Exemplar of a biomarker-based approach to Precision Care for populations with disability. Curr Neurol Neurosci Rep. 2017;17:84. 28929311 10.1007/s11910-017-0791-5

[56] Seif F Khoshmirsafa M Aazami H Mohsenzadegan M Sedighi G Bahar M. The role of JAK-STAT signaling pathway and its regulators in the fate of T helper cells. Cell Commun Signal. 2017;15:23. 28637459 10.1186/s12964-017-0177-y PMC5480189

[57] Saligan LN. Collaborative Framework to advance symptom science: an intramural perspective. J Nurs Scholarsh. 2019;51:17-25. 30375716 10.1111/jnu.12445

[58] Ray M Wallace MK Grayson SC , et al. Epigenomic links between social determinants of health and symptoms: a scoping review. Biol Res Nurs. 2023;25:404-416. 36537264 10.1177/10998004221147300 PMC10404910

[59] Bogie KM Schwartz K Li Y Wang S Dai W Sun J. Exploring adipogenic and myogenic circulatory biomarkers of recurrent pressure injury risk for persons with spinal cord injury. J Circ Biomark. 2020;9:1-7. 33599626 10.33393/jcb.2020.2121 PMC7883629

[60] Bogie K Henzel K Richmond MA Alvarado N. Tissue health biomarkers to predict highest risk individuals for pressure injury recurrence. Arch Phys Med Rehabil. 2018;99:e13.

[61] Schwartz K Henzel MK Ann Richmond M , et al. Biomarkers for recurrent pressure injury risk in persons with spinal cord injury. J Spinal Cord Med. 2020;43:696-703. 31490098 10.1080/10790268.2019.1645406 PMC7534297

[62] De Santa F Narang V Yap ZH , et al. Jmjd3 contributes to the control of gene expression in LPS-activated macrophages. EMBO J. 2009;28:3341-3352. 19779457 10.1038/emboj.2009.271 PMC2752025

[63] Shaw T Martin P. Epigenetic reprogramming during wound healing: loss of polycomb-mediated silencing may enable upregulation of repair genes. EMBO Rep. 2009;10:881-886. 19575012 10.1038/embor.2009.102 PMC2726669

[64] Finelli MJ Wong JK Zou H. Epigenetic regulation of sensory axon regeneration after spinal cord injury. J Neurosci. 2013;33:19664-19676. 24336730 10.1523/JNEUROSCI.0589-13.2013 PMC3858634

[65] Wong JK Zou H. Reshaping the chromatin landscape after spinal cord injury. Front Biol. 2014;9:356-366. 10.1007/s11515-014-1329-8 PMC4280023 25554728

[66] Cashion AK Grady PA. The National Institutes of Health/National Institutes of Nursing Research intramural research program and the development of the National Institutes of Health Symptom Science Model. Nurs Outlook. 2015;63:484-487. 26187087 10.1016/j.outlook.2015.03.001 PMC4507439

[67] Koleck TA Topaz M Tatonetti NP , et al. Characterizing shared and distinct symptom clusters in common chronic conditions through natural language processing of nursing notes. Res Nurs Health. 2021;44:906-919. 34637147 10.1002/nur.22190 PMC8641786

[68] Virolainen SJ VonHandorf A Viel KCMF Weirauch MT Kottyan LC. Gene-environment interactions and their impact on human health. Genes Immun. 2023;24:1-11. 36585519 10.1038/s41435-022-00192-6 PMC9801363

[69] Hunter Revell SM . Symptom clusters in traumatic spinal cord injury: an exploratory literature review. J Neurosci Nurs. 2011;43:85-93. 21488582 10.1097/jnn.0b013e31820c2533

[bibr70-25168657231205679] Lee SE Vincent C Finnegan L. An analysis and evaluation of the theory of unpleasant symptoms. ANS Adv Nurs Sci. 2017;40:E16-E39. 10.1097/ANS.0000000000000141 27525959

[bibr71-25168657231205679] Pickler RH. Advances and challenges in symptom science. Nurs Res. 2020;69:89-90. 32108737 10.1097/NNR.0000000000000416

[72] Kurnat-Thoma EL Graves LY Billones RR . A concept development for the symptom science model 2.0. Nurs Res. 2022;71:E48-E60. 10.1097/NNR.0000000000000605 PMC9640281 35584269

